# Viral Uncoating Is Directional: Exit of the Genomic RNA in a Common Cold Virus Starts with the Poly-(A) Tail at the 3′-End

**DOI:** 10.1371/journal.ppat.1003270

**Published:** 2013-04-04

**Authors:** Shushan Harutyunyan, Mohit Kumar, Arthur Sedivy, Xavier Subirats, Heinrich Kowalski, Gottfried Köhler, Dieter Blaas

**Affiliations:** 1 Max F. Perutz Laboratories, Department of Medical Biochemistry, Medical University of Vienna, Vienna, Austria; 2 Max F. Perutz Laboratories, Department of Structural Biology, University of Vienna, Vienna, Austria; Institut Pasteur, France

## Abstract

Upon infection, many RNA viruses reorganize their capsid for release of the genome into the host cell cytosol for replication. Often, this process is triggered by receptor binding and/or by the acidic environment in endosomes. In the genus *Enterovirus*, which includes more than 150 human rhinovirus (HRV) serotypes causing the common cold, there is persuasive evidence that the viral RNA exits single-stranded through channels formed in the protein shell. We have determined the time-dependent emergence of the RNA ends from HRV2 on incubation of virions at 56°C using hybridization with specific oligonucleotides and detection by fluorescence correlation spectroscopy. We report that psoralen UV crosslinking prevents complete RNA release, allowing for identification of the sequences remaining inside the capsid. We also present the structure of uncoating intermediates in which parts of the RNA are condensed and take the form of a rod that is directed roughly towards a two-fold icosahedral axis, the presumed RNA exit point. Taken together, in contrast to schemes frequently depicted in textbooks and reviews, our findings demonstrate that exit of the RNA starts from the 3′-end. This suggests that packaging also occurs in an ordered manner resulting in the 3′-poly-(A) tail becoming located close to a position of pore formation during conversion of the virion into a subviral particle. This directional genome release may be common to many icosahedral non-enveloped single-stranded RNA viruses.

## Author Summary

Viral infection requires safe transfer of the viral genome from within the protective protein shell into the host cell's cytosol. For many viruses this happens after uptake into endosomes, where receptor-binding and/or the acidic pH trigger conformational modifications or disassembly of the shell, allowing the nucleic acids to escape. For example, common cold viruses are converted into subviral particles still containing the single-stranded positive sense RNA genome; subsequently, the RNA escapes into the cytoplasm, leaving behind empty capsids. We triggered this process by heating HRV2 to 56°C and found that 3′- and 5′-end emerged with different kinetics. Crosslinking prevented complete RNA egress and upon nuclease digestion only sequences derived from the 5′-end were protected. Part of the RNA remaining within the viral shell adopted a rod-like shape pointing towards one of the two-fold axes where the RNA is presumed to exit in single-stranded form. Egress thus commences with the poly-(A) tail and not with the genome-linked peptide VPg. This suggests that assembly and uncoating are well-coordinated to avoid tangling, kinetic traps, and/or simultaneous exit of the two RNA ends at different sites.

## Introduction

Human rhinoviruses (HRV), members of the picornavirus family, genus *Enterovirus*, are the major causative agent of the common cold. Additionally, they play an important role in the exacerbation of asthma, cystic fibrosis, and chronic obstructive pulmonary disease [1]. Similar to other picornaviruses, the species HRV-A, -B, and -C, are composed of 60 copies each of four capsid proteins, VP1, VP2, VP3, and the small myristoylated VP4, arranged on an icosahedral T = 1, P = 3 lattice. The diameter of the particle is roughly 30 nm. The viral genome is a single-stranded RNA molecule of positive polarity, about 7100 bases in length. It carries a covalently linked peptide (VPg) at its 5′-end and a poly-(A) tail of about 70 to 150 bases at its 3′-end [2], [3]. The 5′-nontranslated region is approximately 650 bases in length, highly structured, and involved in cap-independent translation initiation and RNA replication [4].

Minor group rhinoviruses, exemplified by the prototype strain HRV2, bind members of the low-density lipoprotein receptor (LDLR) family including LDLR and LDLR-related protein for entry via clathrin-dependent endocytosis [5]. Once in the endosome, the low pH leads to dissociation of the virus from the receptors as well as to structural changes in the viral capsid [6], [7]; more specifically, the native virion sedimenting at 150S converts into the subviral A-particle sedimenting at 135S and devoid of the internal capsid protein VP4 [8], [9] and exposure of amphipathic N-terminal sequences of VP1 renders it hydrophobic, thus allowing its direct attachment to the inner endosomal membrane [10]. These processes are accompanied by an expansion of the viral shell by about 4% along with the opening of symmetry-related channels. The largest channels are at the two-fold axes, whereas the smaller ones are located near the pseudo three-fold axes and at the base of the star-shaped vertices of the icosahedron [11], [12, and our unpublished resuts]. Finally, the RNA is released through one of these pores, most probably at a 2-fold axis as suggested from cryo-electron microscopy (cryo-EM) image reconstructions of the related poliovirus in which uncoating was induced by heating to 56°C [13], [14]. The final product of this uncoating process is the empty capsid (80S B-particle). Most enteroviruses are believed to undergo similar conformational changes; however, with the exception of minor receptor group rhinoviruses, the process appears to be triggered by receptor binding and possibly assisted by low pH [7], [15]–[17].

These structural modifications can be mimicked, at least partially, *in vitro*. Exposure to pH<5.8 converts native HRV2 preferentially into A-particles whereas incubation at 50°C–56°C in low ionic strength buffers favours conversion into B-particles (empty capsids) [18]. *In vivo*, and in the presence of liposomes *in vitro* [19], both VP4 and N-terminal sequences of VP1 insert into lipid bilayers. They might contribute to formation of a pore connecting the virus interior with the cytosol of the host cell, thus allowing for the transit of RNA in its unfolded form (reviewed in [20], [21]). The necessity for unfolding was suggested by experiments with poliovirus, which demonstrated loss of the intercalating dye Syto 82 during RNA egress [22]. The mechanism of RNA exit is poorly understood. Energy would be required for breaking the hydrogen bonds of the double-stranded regions in the encapsidated RNA genome [23], [24] in order to allow the RNA to thread through an opening only large enough to enable passage of a single strand [12]; however the source of this energy for *in vivo* uncoating is unknown. It appears highly likely that either the poly-(A) tail at the 3′-end or the VPg peptide linked to the 5′-phosphate of the RNA via a tyrosine ester, begins to emerge from the virion since other modes might be unproductive (e.g., simultaneous exit of both ends would be expected to impede complete uncoating and thus to be abortive). Directionality of this process may indicate that the RNA adopts a defined conformation inside the viral shell suggesting a well-organized process of assembly and uncoating. Here, we show that RNA exit does indeed occur in a specific and ordered manner, starting from the 3′-end.

## Results

### Incubation of HRV2 at 56°C releases 5′ and 3′ sequences of the viral RNA with different kinetics

**Figure 1:**
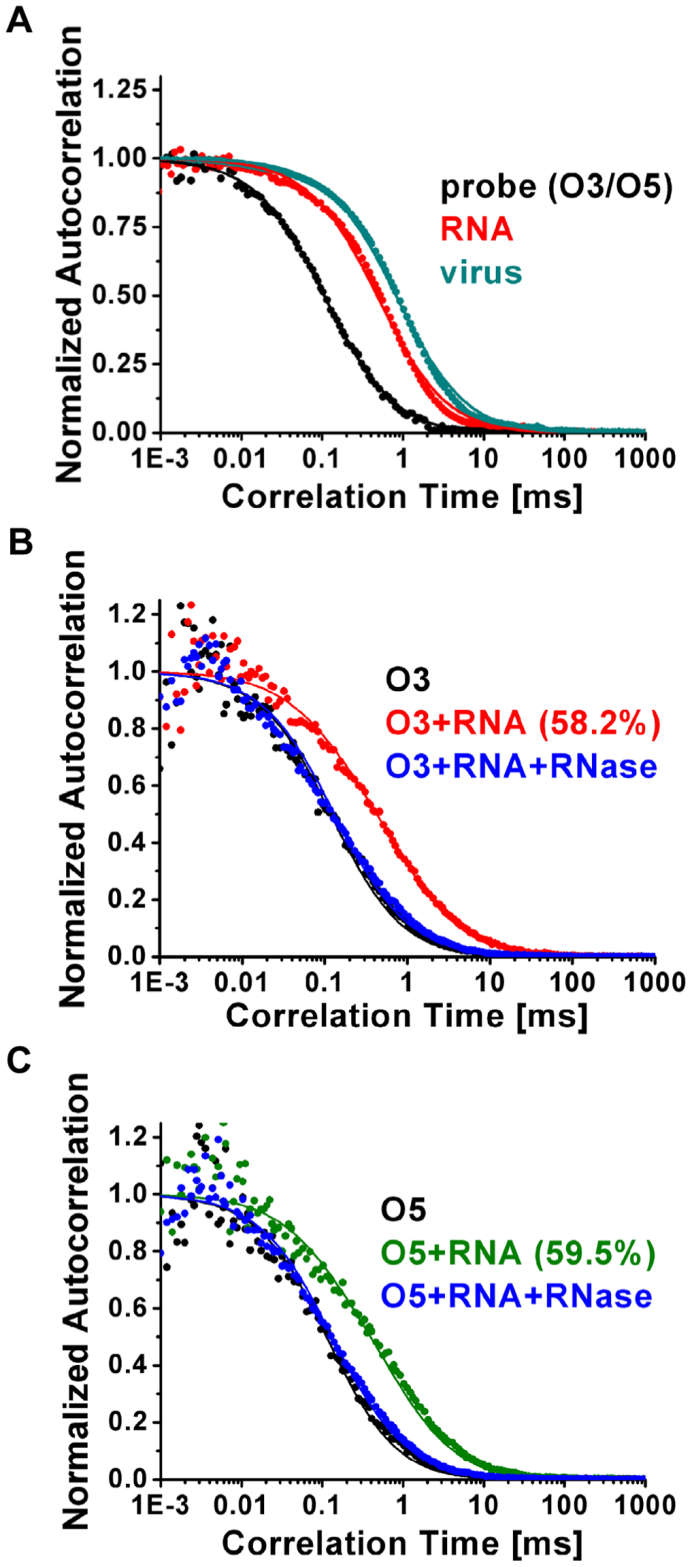
Normalized autocorrelation curves for HRV2, viral RNA, and oligonucleotides obtained by fluorescence correlation spectroscopy. (A) DyLight 488-labeled HRV2 (cyan), YOYO-1 iodide509-labeled *in vitro*-transcribed viral RNA (red), and the labeled oligonucleotides (DyLight 488-labeled 3′-oligo-nucleotide and FAM-labeled 5′-oligo-nucleotide had the same value; both in black) exhibit different diffusion times. The respective oligonucleotides (B and C, black) were separately hybridized to *in vitro*-transcribed viral RNA (3′ red and 5′ green) and measured before and after digestion with RNase A/RNase H (blue), respectively. Note that essentially the same results were obtained with RNA released from virions by heating to 56°C for 20 min. Dots, measured data; continuous lines, one-component (A, and black lines in B, C) and two-component fit (colored lines in B, C; contribution of the 2^nd^ component calculated from the fit is given in %). Note the complete overlap of the curves corresponding to the free oligonucleotides and to the RNase-digested samples.

Fluorescence correlation spectroscopy (FCS) allows measuring the diffusion time, and thus determining the diffusion coefficient, of fluorescent molecules at very low concentrations (down to picomoles/l) in very small sample volumes (down to femtoliters) [25], [26]. Binding of a small labeled molecule to a substantially larger one gives rise to fluorescent complexes that diffuse slowly [27]. Deconvolution of the autocorrelation function of the free and the complexed component allows for calculation of their respective fractions present in the mixture. For instance, recombinant very-low density lipoprotein receptor fragments (M_r_ = 12 to 80 kD) were labeled with Cy3 at their N-termini and the change of their diffusion coefficient upon binding to HRV2 (M_r_ = 8.5 MD) was monitored [28]. Resolving the fraction of free and virus-bound receptor fragments (at various virus concentrations) allowed determination of the binding constants. Here we used fluorescently labeled oligonucleotides complementary to regions near the 3′ and the 5′-ends of the RNA molecule for hybridization to detect sequences becoming accessible during viral uncoating. In control experiments shown in Fig. 1A to C, the diffusion coefficients (Table 1 and 2) were determined for each of the three reaction components (see Methods section). Fig. 1A shows that the autocorrelation functions of fluorescent oligonucleotide probe, YOYO-labeled *in vitro*-transcribed HRV2 RNA, and Dylight-labeled virus are sufficiently different to allow for determination of their relative concentrations in the mixture; attempts at determining the autocorrelation function for virus with partially-released RNA were hampered by the heterogeneity of the samples obtained after the crosslinking step that was necessary for halting complete egress upon heating (see below). As expected, the values were generally somewhat lower than those for native virus, however, they varied considerably. For this reason, all of the calculations below were carried out using the diffusion coefficient of the native virus instead. Further control experiments (Fig. 1B and C) demonstrated a rightward shift of the autocorrelation curve of the 5′ and 3′end-specific oligonucleotides upon binding to *in vitro*-transcribed viral RNA and a reversal on digestion with RNases. The diffusion coefficient was virtually identical for viral RNA obtained by heating HRV2 to 56°C for 20 min and *in vitro*-transcribed RNA (not shown). Thus, the slightly different size of the poly-(A) tail, as well as the small VPg that is absent from *in vitro*-transcribed RNA, had no significant impact on the diffusion properties. The measured and calculated diffusion coefficients of the probes, the free RNA, and the virus are summarized in Table 1 and the values for the two different probes, either free or hybridized to viral RNA, prior and after digestion with RNases, are shown in Table 2.

**Table 1:** Diffusion coefficients (D) of labeled free oligonucleotides (probe), YOYO-1 iodide509-labeled *in vitro*-transcribed RNA, and DyLight 488-labeled virus.

	D (10^−12^) m^2^/s	
	Measured	Estimated
**probe** 1	73.9±6.4	65*<probe<180+
**RNA**	16.5±1.6	7.5†<RNA<28+
**HRV2**	9.6±0.3	HRV2<15+

The mean values and the standard deviation were calculated from at least 5 separate experiments with at least 20 repetitions each.

1 Mean of 3′ and 5′ probes.

Diffusion coefficients calculated assuming different shapes:

+ sphere;

* rod;

† random coil. Note that virus with partially extruded RNA was not available in pure form and thus its diffusion constant could not be measured. The value of native virus was used instead in all experiments.

**Table 2:** Diffusion coefficients of free probes, probes hybridized to RNA, and probes hybridized to RNA followed RNaseA/RNaseH digestion.

	5′		3′	
	D (10^−12^) m^2^/s	2^nd^ comp.	D (10^−12^) m^2^/s	2^nd^ comp.
**Probe**	84.2±1.6		80.3±1.7	
**probe+RNA**	15.0±1.9	59.5%±8.1%	13.6±2.0	58.2%±6.1%
**probe+RNA+RNase**	78.4±3.2		83.7±2.8	

The fraction hybridized was determined from a two-component fit of the autocorrelation functions. Note that the slight difference from the values in Table 1 is due to the use of a different buffer necessary for RNase treatment and that RNA (Table 1) and probe bound to RNA (Table 2) had virtually identical diffusion coefficients.

**Figure 2:**
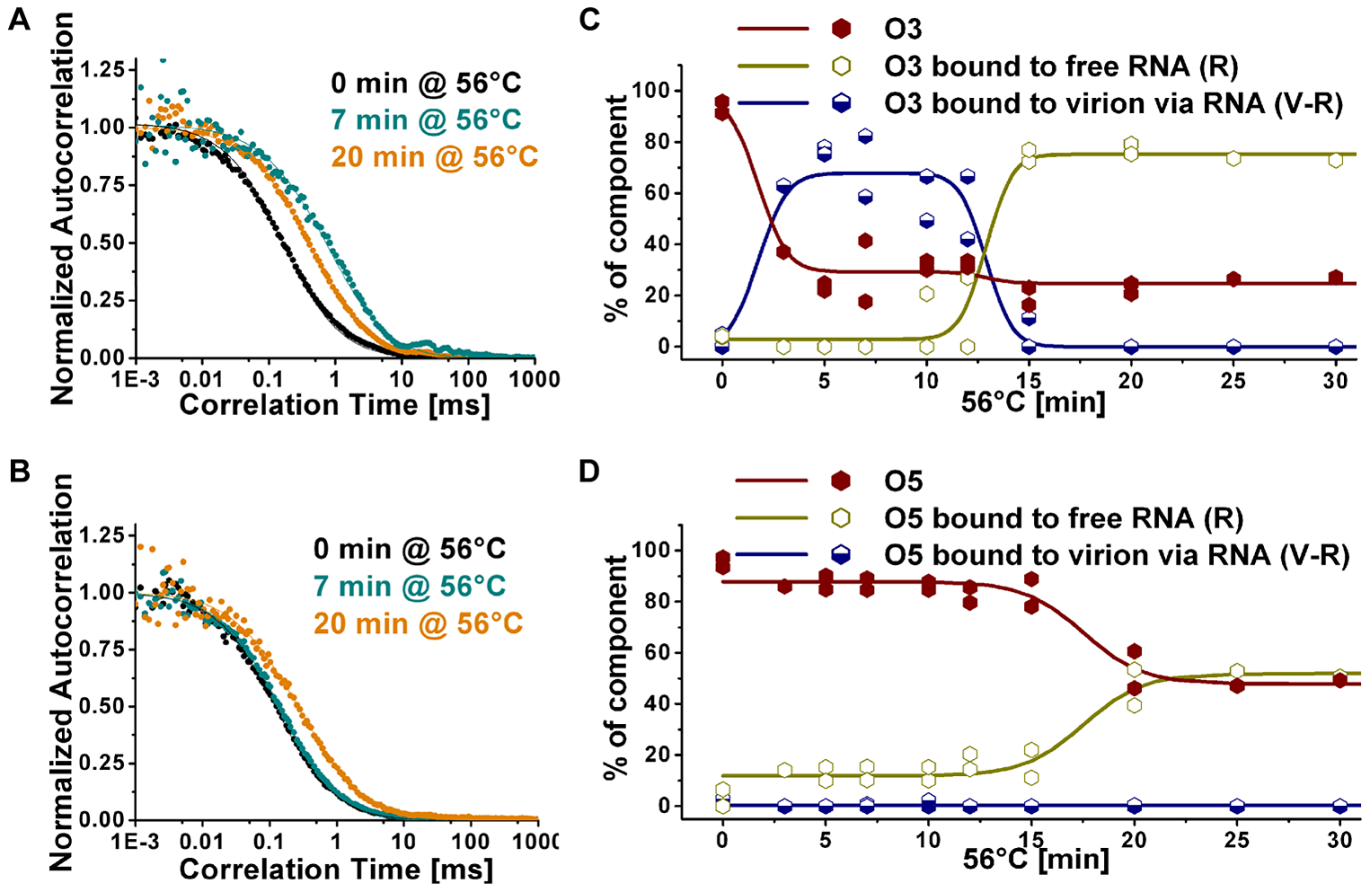
RNA sequences at the 3′ and the 5′-ends become accessible for hybridization with different kinetics. HRV2 was incubated at 56°C for 0, 7, and 20 min. Dylight 488-labeled oligonucleotide specific for 3′ sequences (A) and 5′ sequences (B) was added and the autocorrelation function was measured (C, D). Identical experiments were carried out again with additional time points. The percentage of free oligonucleotide (red), oligonucleotide hybridized to free RNA (ochre) and oligonucleotide hybridized to RNA connected to the virus (blue) was calculated for each time point by employing a three-component fitting procedure (symbols). For better appreciation, the respective values for each constituent (free oligo, oligo bound to RNA, and oligo bound to RNA partially extruded from the virion) were fit by a sum of Boltzmann functions (lines). Data points are from two independent experiments. Note that repeated experiments showed the same trend over time, but there were differences in background and in the absolute values of the measurements.

HRV2 was incubated at 56°C in the presence of the respective probes for the times indicated in Fig. 2 and cooled on ice; the autocorrelation function of the oligonucleotides hybridized to cognate-accessible RNA sequences was then measured at ambient temperature. Cooling halted further exit of the RNA when returned to room temperature, whereas RNA egress continued at a markedly reduced rate when this step was omitted (data not shown). For the 3′-specific probe (Fig. 2A), the correlation time was increased at 7 min (due to binding to partially-extruded RNA that was still connected with the protein shell) but decreased at 20 min to a value corresponding to the probe now hybridized to free RNA. Conversely, in case of the 5′-specific oligonucleotide, it remained unchanged at 7 min and increased at 20 min, but did not exceed the value corresponding to free RNA (Fig. 2B).

Measurements were then repeated with additional incubation times. The percentages of free oligonucleotide, oligonucleotide bound to free RNA, and oligonucleotide bound to virus with part of its RNA having become exposed, were obtained by fitting the data to a three-component autocorrelation function using the diffusion coefficients of each constituent obtained in the previous experiment. We assumed that the value for virus and virus with partially-extruded RNA were negligibly different (see above and Table 1). Fig. 2C and D show a clear difference in the time-dependent change of the percentages of accessible 3′ and 5′ ends, free RNA, and RNA attached to the virion. At 3 min incubation at 56°C, the fraction of the free 3′-oligonucleotide (O3) had strongly diminished. This was accompanied by an increase of O3 bound to virus with externalized RNA (V-R). Between 10 and 15 min V-R diminished again, whereas O3 bound to free RNA (R) increased until attaining a plateau. Conversely, as shown in Fig. 2D, the fraction of free 5′-specific oligonucleotide (O5) only started to decrease at about 12 min, with a concomitant increase of RNA-bound O5. Apparently, part of the RNA molecules had been entirely released at that time resulting in the 5′-ends being accessible for hybridization with the probe. The plateau at about 20 min indicates that no further RNA became available for hybridization, suggesting that release was completed. There was no significant change in V-R over the observation time indicating absence of virus with exposed RNA harbouring the sequence complementary to O5 (Fig. 2D). Control experiments showed that RNA exit was insignificant at ambient temperature during the timeframe of the experiment. Nevertheless, cooling to 4°C appeared important, as keeping the sample at room temperature for more than 10 min resulted in aggregation (as indicated by the appearance of fluorescent species with very long diffusion times; not shown). Whereas hybridization to O3 plateaued at about 75%, hybridization to O5 only reached about 50% possibly due to the higher number of potential binding sites for the former (the poly-(A) is between 50 and 200 nucleotides long whereas the region complementary to O5 is unique; see also Materials and Methods).

### Psoralen crosslinking halts RNA release

In order to confirm and complement the FCS results, we determined the segment of the RNA remaining inside the virion. This was achieved by halting the uncoating reaction when only part of the RNA had left the virion and removing the exposed RNA by RNase digestion. Since psoralen crosslinking readily abrogated infectivity of the related poliovirus [29], we reasoned that it may impede egress of the entire genomic RNA by preventing complete unfolding of double-stranded regions [30] during uncoating. This would lead to the accumulation of structures intermediate between full 135S particles (A-particles with genomic RNA not yet released) and empty capsids to levels high enough to allow for their characterization.

**Figure 3:**
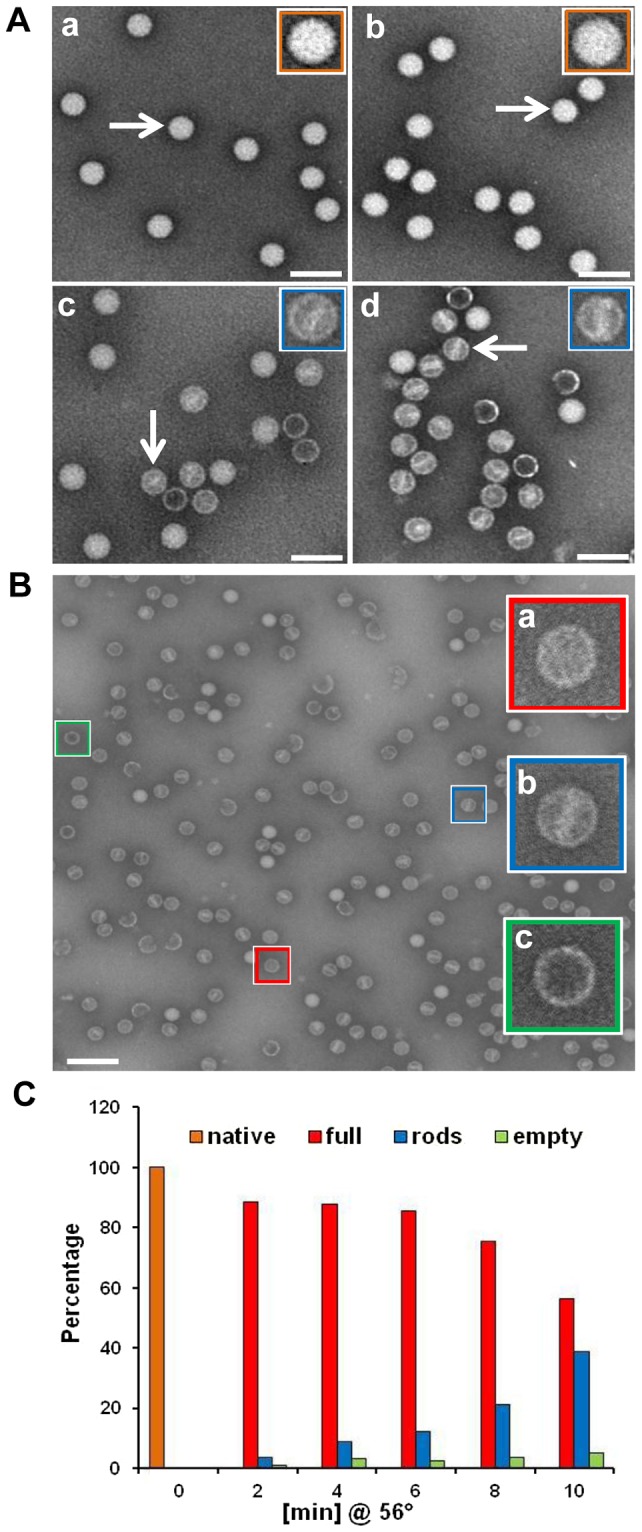
Subviral particles containing rod-like density accumulate on incubation of psoralen UV-crosslinked virions at 56°C. A) Electron micrographs of phosphotungstate-stained HRV2 without a) and with b) psoralen UV crosslinking. Panels c) and d) samples were treated as a) and b) but heated to 56°C for 10 min. B) Incubation of psoralen UV crosslinked HRV2 as in Ad) resulted in the generation of various subviral particles. Examples selected visually are depicted in the insets. C) Particles representative for the examples in B) found at various incubation times were visually identified and counted on micrographs (number of particles counted at 2 min, 6,672; 4 min, 6,026; 6 min, 10,847; 8 min, 5,000; 10 min, 7,470; total count at time 0 was taken as 100% native). The mean of three separate experiments is shown. Since it is not possible to reliably distinguish native virus from full 135S A-particles by this method, we conjecture that particles classified as ‘full’ might also include virions that are still in the native conformation. Note that some broken particles are also visible in the micrographs. Bar, 100 nm.

Small molecules such as N-acetyl-aziridine [31] and Ribogreen [32] can diffuse into native HRV at physiologic temperature due to ‘breathing’ and bind to the RNA, rendering it fluorescent. Control experiments showed that incubation of purified HRV2 with 8-Methoxypsoralen (8-MOP) for 4 h at 37°C followed by irradiation at 365 nm led to an approximately 90% loss of viral infectivity (data not shown), indicating that this compound was also able to diffuse into the virion. We therefore assessed the effect of this treatment on the integrity of the virus and its subviral particles formed on incubation at 56°C by negative staining electron microscopy (EM). As seen in Fig. 3, psoralen crosslinking did not change the morphology of native virus (compare Fig. 3Aa and 3Ab). When such samples were heated to 56°C, both native virus and crosslinked virus were converted into particles possessing an internal density with a rod-like appearance, but to a remarkably different extent. Without crosslinking, their proportion was low (Fig. 3Ac), but it substantially increased upon crosslinking prior to heating (Fig. 3Ad). Conversion from full to empty capsids was apparently halted at a stage where some RNA was still remaining inside the virion (with part of it assuming a rod-like shape) and relatively few empty particles were observed (Fig. 3B). The time-dependent formation of these ‘rod-particles’ from psoralen-crosslinked virions was assessed by visual inspection of the micrographs and counting. The result of a representative experiment is depicted in Fig. 3C. Native virions (present at t = 0) and ‘full-looking’ particles (HRV2 remaining native plus A-particles present at t>0) decreased over time with a concomitant increase of ‘rod-particles’ and a minority of ‘empty-looking’ particles (see inset in Fig. 3B for typical examples of these particles). Presumably, egress of the RNA (single-stranded) through the pore in the viral shell was arrested as soon as a crosslink was encountered. Apparently, the RNA adopts the form of a rod as a consequence of heat-triggered partial release. After 10 min, about 39% of the virus had converted into particles containing condensed RNA, indicated by either a typical ‘rod’ or a dot appearance in the center, depending on the orientation of the virion with respect to the plane of the grid. There were ∼5% empty particles and a remainder of 56% ‘native-like’ (i.e. ‘full-looking’) virions. Further extension of the incubation time at 56°C led to accumulation of viral debris, suggesting that prevention of ordered uncoating may result in (partial) disruption of the virion (see Suppl. Material, Fig. S2).

### Three-dimensional structure of the ‘rod-particles’

**Figure 4:**
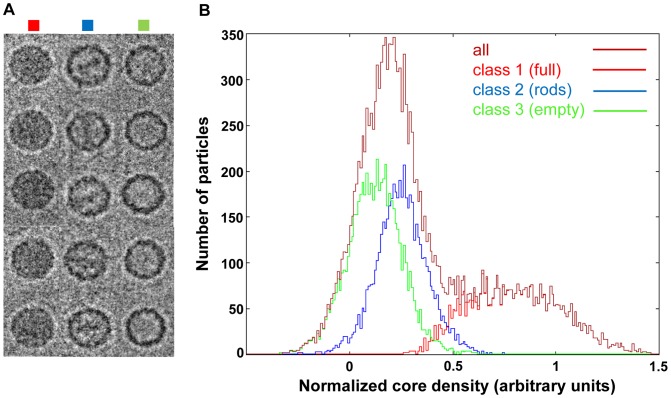
Cryo-electron microscopy image analysis of subviral HRV2 particles obtained on heating psoralen UV crosslinked virions. A) Examples of micrographs of particles visually classified as full (left column), ‘rod-containing’ (middle column), and empty (right column). B) Distribution of the relative RNA density in the individual particle images. Core density (r = 0–113 Å) was related to part of the density of the protein shell (r = 113–143 Å; see Fig. S1) for all particle images (all) and the classes obtained by ML3D-classification. Class1 (full), class2 (rods), class3 (empty) relate to the 3DR shown in Fig. 5.

**Figure 5:**
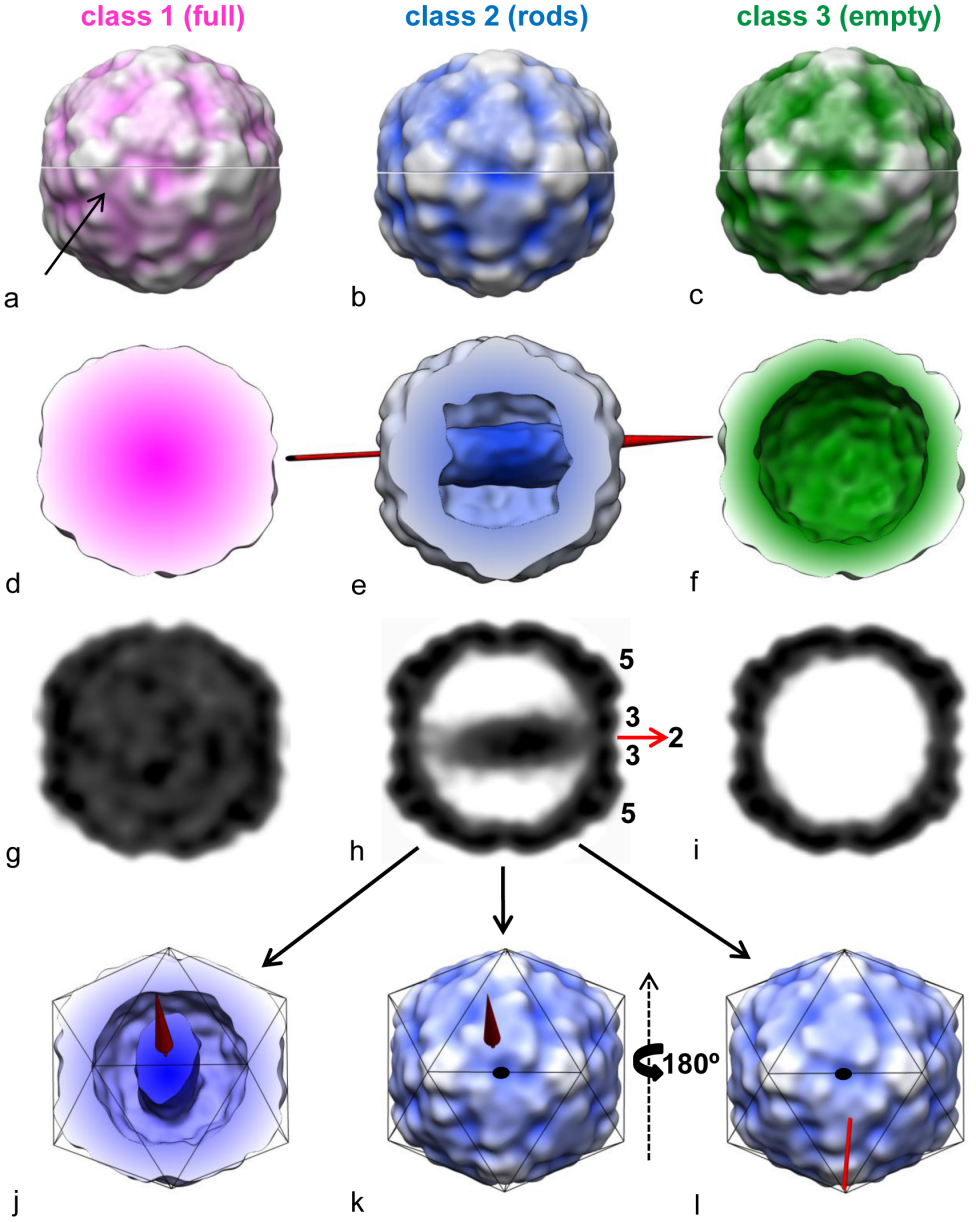
Cryo-electron microscopy image reconstruction of class1, 2, and 3 as obtained by maximum likelihood 3D-classification of psoralen UV crosslinked and heated virions viewed down a 2-fold axis. (a)–(c), radially color-coded volumes obtained on 3DR imposing icosahedral symmetry (upper half) or without imposing symmetry (lower half); (d) and (f), transverse central sections; (e) for better appreciation of the internal rod-like density, the particle was cut open at about 6 nm above the center of the virion; (g)–(i), central slabs (∼3.8 Å thick); in (e) and (j)–(l), class2 particles are shown with an arrow along the longitudinal axis of the rod indicating the orientation of the internal density with respect to the icosahedral symmetry. Note that the views are on different 2-fold axes. Rotation of the model (k) by 180° around the y-axis indicates that the ‘rod’ contacts the inner face of the virion shell at positions close to a 2-fold and a 3-fold axis at roughly opposite sides. All volumes are depicted with sigma = 1 above the mean density. In (h) the positions of 2-fold (red arrow), 3-fold, and 5-fold axes are indicated. In (a), an obvious deviation from symmetry is indicated with a small black arrow.

In order to exclude the possibility that the rod-like density inside the subviral particles observed in negative stain EM was a staining artefact, we also performed cryo-EM analysis. Samples were prepared as above but applied to microscope grids with holey carbon film, frozen, and images were recorded. As seen from the representative images in Fig. 4A, particles appearing full, containing rod-like density, and apparently empty particles were again observed. The dataset of downscaled particle images (64×64 pixels) was submitted to maximum likelihood 3D (ML3D) classification [33], [34] imposing three classes and particle images assigned to each class were analyzed individually for their relative RNA content by relating the density in the core (mainly corresponding to the RNA) to the density corresponding to the protein shell. Fig. 4B shows that the distribution of the RNA content in the entire dataset was bimodal with a peak and a broad shoulder. Separate analysis of the images corresponding to the respective classes yielded three partially-overlapping peaks; one representing high, but considerably variable, RNA content (class1), and two with intermediate (class2) and low (class3) RNA content, respectively. The reconstructions were then further refined by using particle images (now at 128×128 pixels) corresponding to the three respective classes; icosahedral symmetry was imposed (top half of the models) or not (lower half of the models). These are depicted in Fig. 5(a)–(c) as radially color-coded surfaces with the view down a 2-fold axis, as transverse sections (d)–(f), and as central planes (g)–(i). Despite the lower resolution of the asymmetric reconstruction (24 Å, 22 Å, and 22 Å, respectively), as compared to 14 Å, 13 Å, and 13 Å obtained when imposing symmetry, the icosahedral shape of the virus was quite well-defined in all three classes when rendered at a contour level of sigma = 1 above the mean density. Nevertheless, comparing the volumes reconstructed with and without imposing symmetry, the (almost) full particle had asymmetric features, whereas empty and ‘rod-containing’ particles were remarkably symmetric (e.g. see Fig. S2B and compare the five-fold axes on the left of the class1 particle in Fig. 5a, black arrow indicating the distortion). The viral shell showed no obvious deviation from symmetry with respect to the orientation of the rod (compare also to Fig. S2). The spherically averaged radial density plots (Fig. S1) established that the three models had similar diameters. All particles were by 4% larger than native virions; therefore, the ‘full’ particles are probably more similar to subviral A-particles than to native virions but contain variable amounts of RNA (Fig. 4B).

Determination of the long axis passing through the center of mass of the rod-like density allowed estimation of its approximate orientation with respect to the icosahedral axes of the virion. Fig. 5(j)–(l), show that the rod does not traverse the center of the virion but is slightly shifted aside. As a result, it contacts the protein shell at a position close to a 2-fold axis and close to a 3-fold axis on roughly the opposite face.

As anticipated from visual inspection of the micrographs (Fig. 4A), extending the number of classes in the ML3D analysis from 3 to 10 revealed a more heterogeneous dataset (Fig. S2). Four classes that were each populated by less than 5% of the total particle images corresponded to damaged or heavily distorted virions (not shown), one class was represented by (almost) full virions, 2 classes corresponded to (almost) empty particles with one of them lacking a pentamer, and 3 classes constituted particles with significant ‘rod-like’ density. In keeping with the heterogeneity of the density of the rod seen in Fig. 4A, the three latter classes contained rods of different shape. Upon rendering at sigma = 1.7 (class2), sigma = 2.0 (class3), and sigma = 1.5 (class4) all showed that the rod was contacting the inner face of the capsid close to a 2-fold axis and extending to the opposite side. Despite employing high underfocus we were unable to visualize any RNA outside of the particle. It is possible that traces of RNase remaining from viral purification (see Methods section) digested exposed RNA under the conditions of sample preparation employed for electron microscopy.

### Capillary electrophoresis demonstrates exposure of RNA in crosslinked heated subviral particles

**Figure 6:**
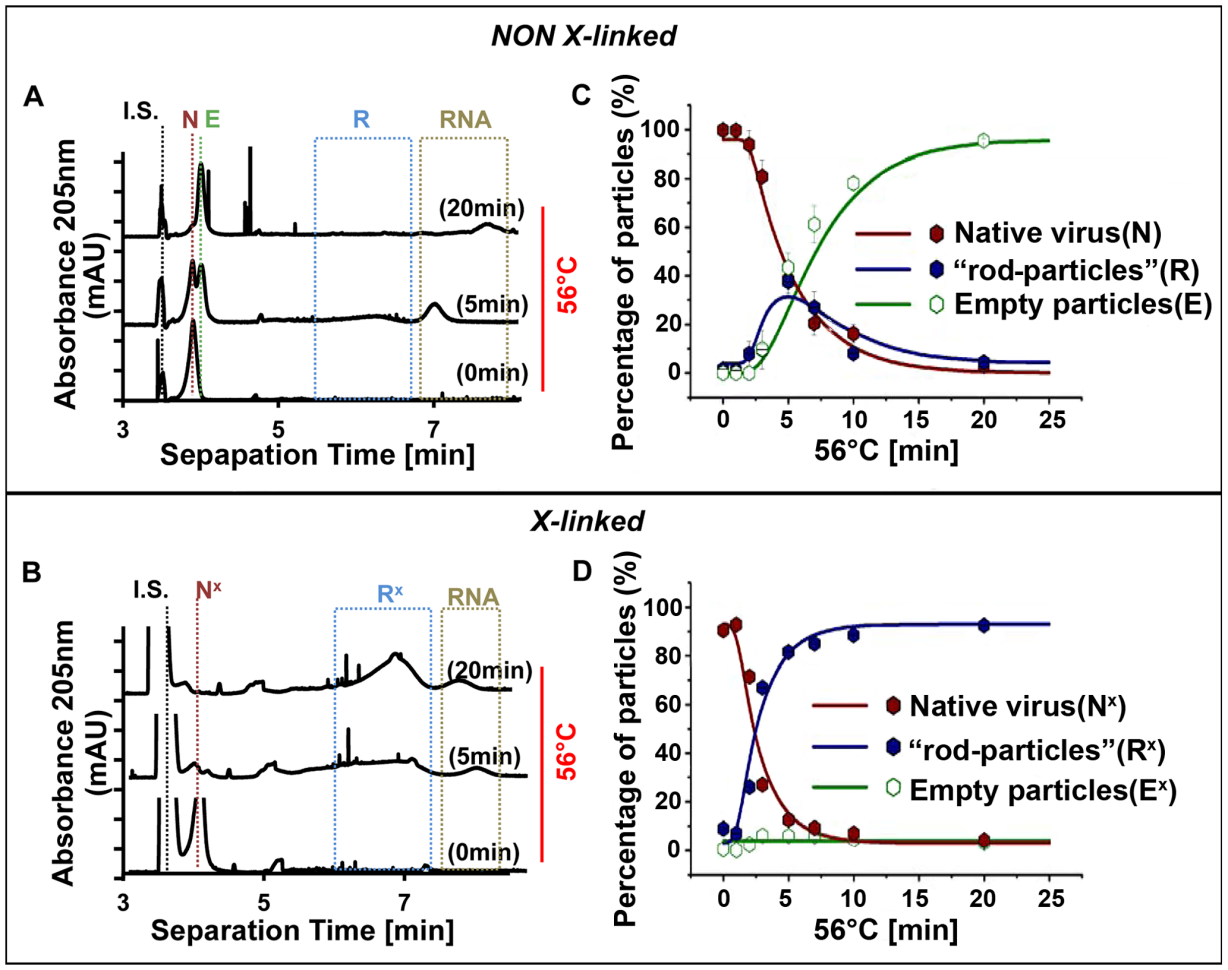
Capillary electrophoretic analysis and quantification of (sub)viral particles obtained on incubation at 56°C. HRV2, non-crosslinked (A) and crosslinked (B), was incubated at 56°C for the times indicated. Note that about 50% and 100% of all non-crosslinked native virus (N) was converted into empty particles and free RNA within 5 min and 20 min, respectively. For crosslinked virus (N^x^) the conversion did not proceed beyond RNA-containing intermediates (R^x^). Nevertheless, some free RNA, presumably with different degrees of degradation as reflected in different migration behaviour, was also seen. Only 3 time points are shown for clarity. In (C) and (D), the three components present in these samples were quantified including more time points. Native virus (crosslinked or non-crosslinked), red; intermediate particles including ‘rod-particles’, blue; empty particles, green. The presence of RNA was ascertained by extinction at 260 nm (not shown but compare to Fig. 7). IS, internal standard; DMSO (3.5 µM). Weiss and colleagues [36] found mobilities [in 10^−9^ m^2^/Vs] of −6.6 for native HRV2, of −8.2 for empty particles, and of −17.1 for intermediate particles. Whereas the present values for native and empty particles were almost identical with the ones determined in this previous work, the intermediate particles migrated as a very broad peak with mobilities between −19.2 and −30.2×10^−9^ m^2^/Vs suggesting that they carry RNA segments of different lengths externally. Continuous lines are derived from modelling the time-dependent equilibrium concentrations of the components with GEPASI [48].

To further assess the nature of these subviral particles, crosslinked HRV2 was incubated at 56°C for different times and subjected to capillary electrophoresis (CE) in the non-ionic detergent Thesit [35]. Native virus, A- and (empty) B-particles were identified according to their electrophoretic mobility as described previously [36]. As seen in Fig. 6A, non-crosslinked native virus used as a control (N) quickly converted into empty capsids (E; to about 50% in 5 min and to almost 100% in 20 min under these particular conditions) whereas crosslinked virus (N^x^) gave rise, almost exclusively, to particles migrating as a broad peak with a substantially increased migration time (R^x^; Fig. 6B). Presumably, as a consequence of incomplete uncoating, R^x^ carry less than the entire complement of the RNA inside the shell, but the remainder is exposed, thereby modifying the surface charge-to-size ratio relevant for the migration behaviour. For quantification, samples were incubated as above, but collected at various time points and analysed. The data are summarized in Fig. 6C and D. The percentage of both non-crosslinked and crosslinked native virus decreased with increased incubation time at 56°C. For the non-crosslinked sample, a small transient increase in the base-line corresponding to heterogeneous RNA-containing intermediate particles (R) was observed; on further heating, these converted completely into empty capsids (E). Conversely, crosslinked virus transformed virtually entirely into such heterogeneous intermediate particles (R^x^). The concentration of empty particles was below the detection limit. Note that R and R^x^ likely include intermediate particles with and without the typical ‘rod-like’ density (as in Fig. 3, 4, and S2), since the conformation of their RNA core is not expected to impact on their migration behaviour in capillary electrophoresis.

**Figure 7:**
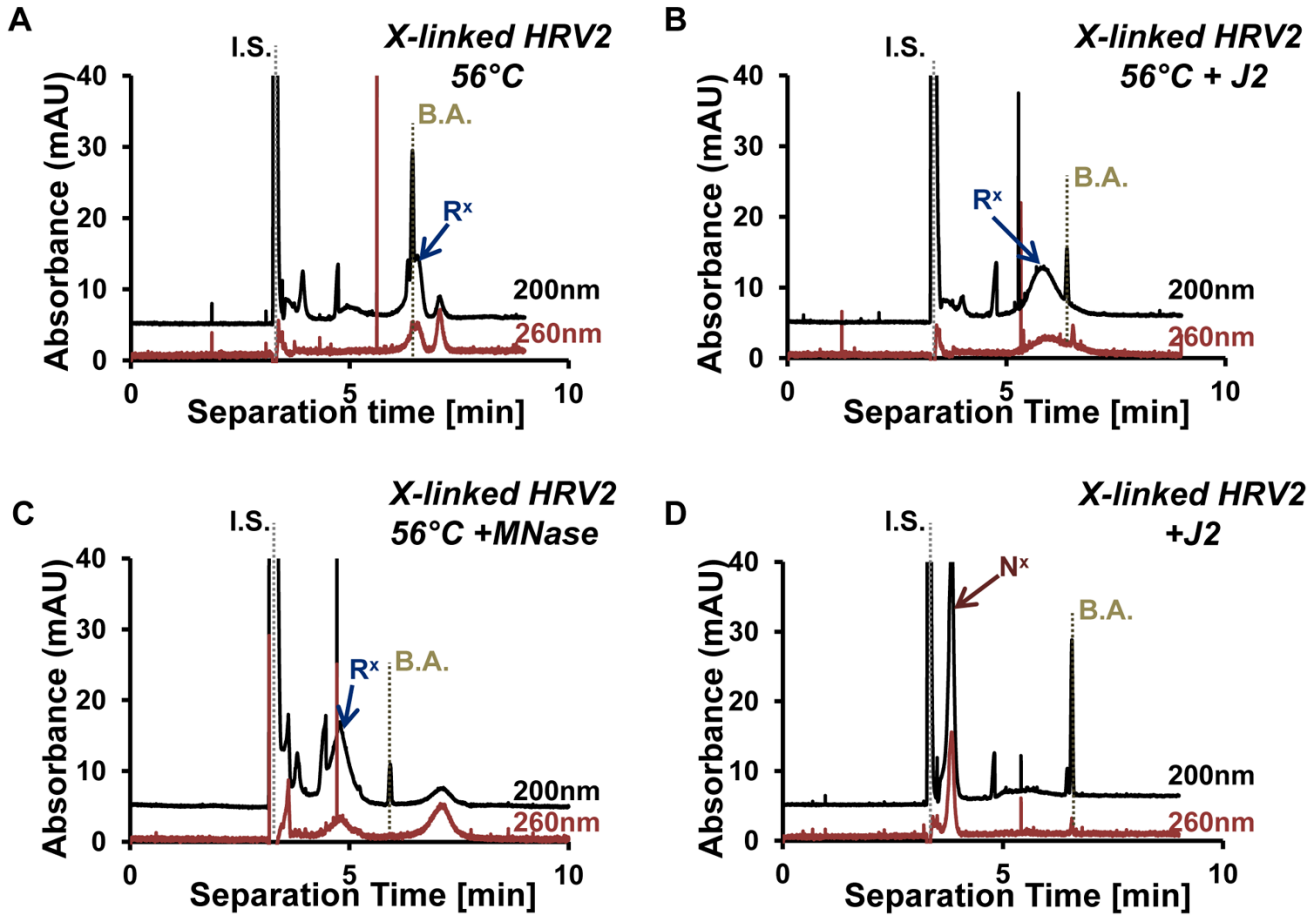
Capillary electrophoresis of crosslinked HRV2 heated to 56°C for 10 min reveals partial exposure of the RNA. A) Control; without antibody. B) As in A, but incubated with mAB J2 directed against dsRNA. C) As in A, but incubated with micrococcal nuclease. D) As in B, but without heat treatment. Note that the antibodies did not bind to the control sample kept at room temperature (D), as the crosslinked virus had the same electrophoretic mobility as native virus [36]. All subviral particles contained RNA as seen from extinction at 260 nm (values multiplied by 3 for better appreciation). Internal standards, DMSO (I.S.) and benzoic acid (B.A.). Blue arrow, uncoating intermediate; red arrow, intact virion.

In order to confirm the presence of exposed RNA in HRV2 subjected to crosslinking and heating as above, the resulting particles were incubated with mAB J2, a monoclonal antibody specifically recognizing dsRNA [37] and again analyzed by CE (Fig. 7). There was a clear shift of the peak related to R^x^ in the presence of the antibody (compare peaks marked with a blue arrow in Fig. 7A and B to the second internal standard, benzoic acid; B.A.). When the sample in Fig. 7A was treated with micrococcal nuclease (MNase), the peak shifted to the position corresponding to 135S particles (blue arrow in Fig. 7C), which have no exposed RNA [36]. Without incubation at 56°C, the crosslinked virus exhibited the electrophoretic mobility of native HRV2 [36] (red arrow) regardless of the presence of the antibody (Fig. 7D). These experiments confirm that only part of the viral RNA can be released in the presence of psoralen crosslinks. This RNA remains connected to the subviral particle, refolds externally, and is therefore detected by dsRNA-specific antibodies.

### Detection of the protected RNA-end by RT-PCR

**Figure 8:**
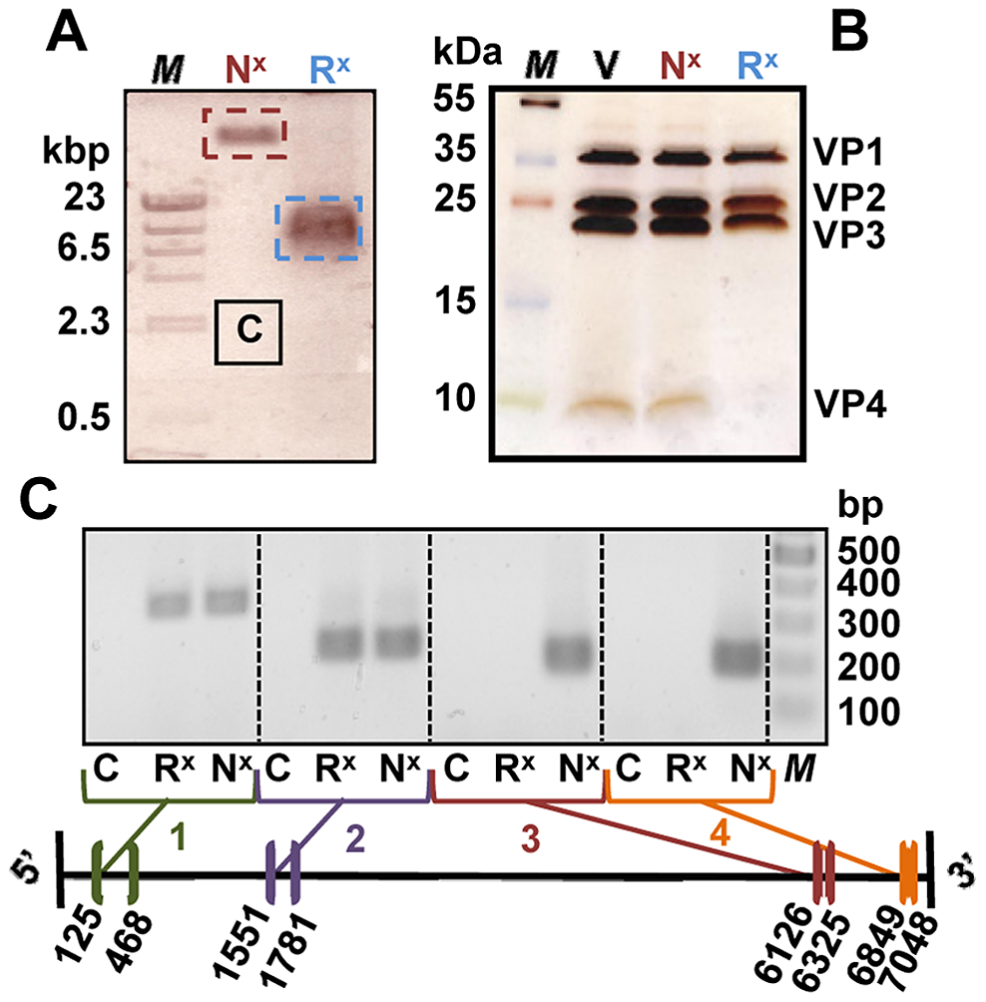
Viral RNA sequences close to the 5′-end are protected against RNases by the protein shell of subviral particles. A) HRV2 was subjected to psoralen UV crosslinking and electrophoresed on a native 0.7% agarose gel without heating (N^x^) and after heating to 56°C for 10 min and incubated with micrococcal nuclease at 37°C for 20 min (R^x^). RNA was identified by ethidium bromide staining and the bands were cut out. B) Aliquots of the excised bands were boiled in reducing sample buffer and the proteins were separated on a 12–20% gradient SDS-PAGE gel followed by silver staining. V, untreated HRV2 used as a control; M, markers. C) RNA was extracted from aliquots of the gel pieces and subjected to RT-PCR using primer pairs complementary to sequences at the positions indicated in the scheme at the bottom. RNA bracketed by the sequences of the respective oligonucleotide pairs was revealed by amplification followed by agarose-gel electrophoresis and staining with ethidium bromide and correlated with the bands of marker DNA run on the same gel.

Crosslinking followed by incubation at 56°C for 10 min resulted in subviral particles that carried part of the viral genome exposed to the outside (see above). Therefore, we determined which part of it remained inside the viral shell and thus protected from RNases. ‘Rod-particles’ were prepared as above at 56°C, accessible RNA was digested with MNase, the nuclease was inactivated with EGTA, and the sample subjected to native agarose gel electrophoresis followed by staining for RNA. As seen in Fig. 8A, crosslinked virus that had not been heated (control; N^x^) showed a well-defined band close to the top of the gel. In contrast, the sample that had been heated and incubated with MNase migrated further (R^x^). The respective bands were cut out and analyzed for the presence of proteins by SDS polyacrylamide gel electrophoresis followed by silver staining (Fig. 8B). The band stemming from crosslinked but not heated virus (N^x^) contained VP1–VP4, whereas crosslinked, heated, and MNase-treated virus (R^x^) only contained VP1–VP3 indicating that it had been converted into subviral 135S A-particles devoid of VP4. RNA was extracted from the remaining part of the gel slices, reverse transcribed and the cDNA amplified with two primer pairs each, selected to produce fragments derived from the 3′ and the 5′-ends, respectively (see scheme on the bottom of Fig. 8C). Based on the gel analysis it becomes clear that cDNA was exclusively amplified from the 5′-end, indicating that at least about 1000 nucleotides of the RNA, including the extreme 3′-end, were lost due to digestion whereas at least one fourth of the genome remained inside the shell. This comprises roughly 35% of the total RNA estimated to be present in the subviral particles including ML3D class1 and class2 comigrating in CE (see Methods section). Control experiments showed that *in vitro*-transcribed RNA and RNA extracted from band N^x^ in Fig. 8A yielded the same fragments at a ratio of about 1∶1 although the crosslinking reduced the overall yield of all fragments to some extent. This suggests that the crosslinks were introduced in a random fashion. A sample of the gel cut at position ‘C’ (Fig. 8A, control) was treated exactly as the other bands, verifying that the amplification reaction did not produce any signal-excluding contamination.

## Discussion

Because of its appealing and suggestive nature, schemes of enterovirus uncoating have long depicted the RNA as exiting at one of the five-fold axes; however, this model was never supported by experimental evidence. Exit at the 5-fold axis was brought into question by results of cryo-EM analysis of intermediate uncoating states of poliovirus [38], and of metastable complexes between HRV3 and a soluble form of its receptor ICAM-1 [39]. These data suggested that structural changes led to thinning of the viral shell at the canyon floor pointing to the probable exit of the N-terminal extension, and even of the RNA, at or near this site. The original model was finally challenged by cryo-EM data of poliovirus heated to 56°C. In these more recent studies, external density was detected close to a two-fold axis and this external density was interpreted to be exiting RNA [13], [14]. This view was supported by the X-ray analyses of the empty capsid of HRV2 [12] and of the converted natural empty particle of EV71 [40], demonstrating that the transition from native virion to empty capsid is accompanied by the opening of channels at the two-fold axes. Additionally, the ‘plug’ of the ß-cylinder built from five copies of VP3 below the vertex at the five-fold axis was found to be virtually unchanged, blocking the small opening seen from the outside. The holes at the two-fold axes are now believed to be exit points for the genomic RNA of enteroviruses.

**Figure 9:**
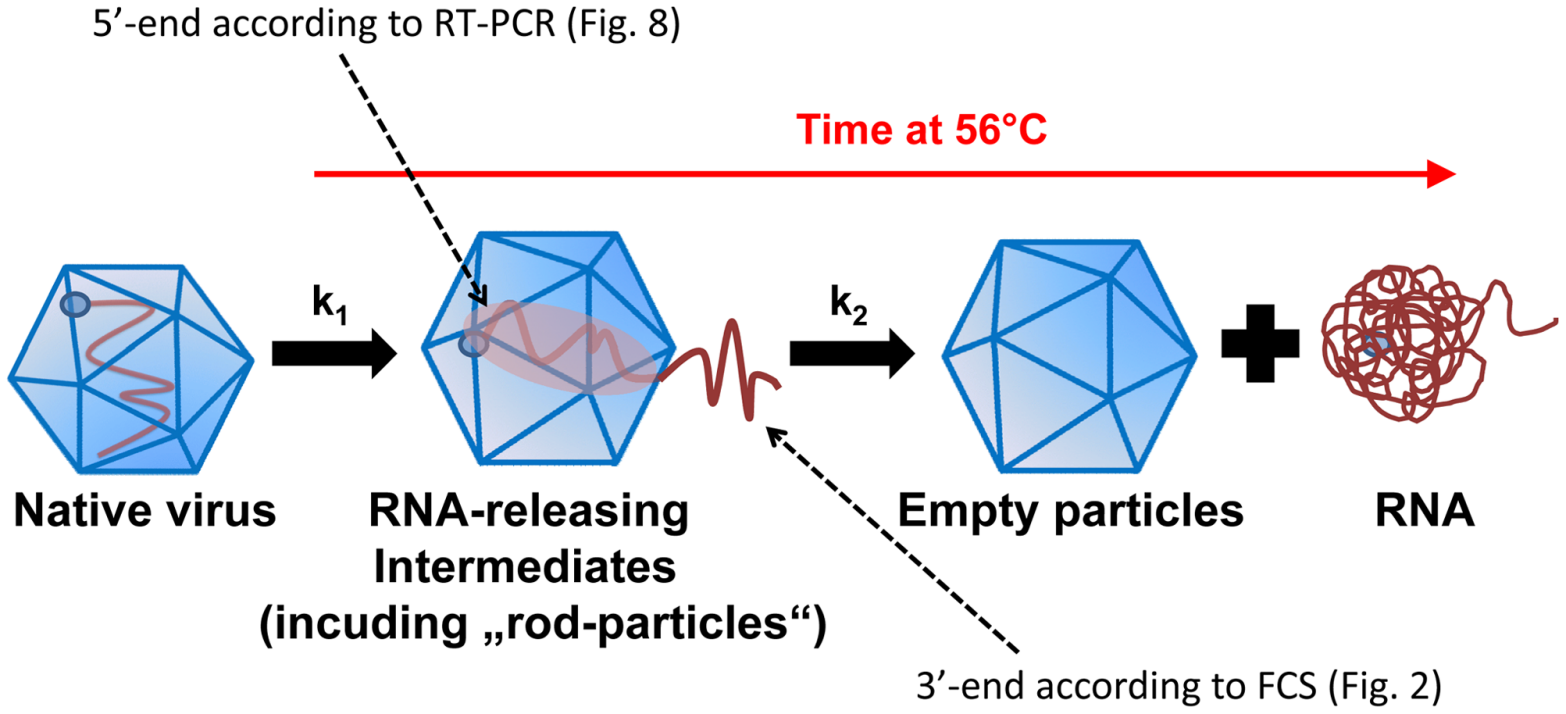
Schematic representation of the time-dependent conversion (with rate constant k1) of native virus to intermediate particles (that are in the process of RNA release and include ‘rod-particles’) and of such intermediates particles further to empty capsids (with rate constant k2). Intermediates with partially-released RNA (with diffusion properties similar as native virus) and free RNA are distinguished by FCS (Fig. 1) and their relative amounts as a function of the incubation time at 56°C of native virus were determined by FCS using oligonucleotides hybridizing to sequences close to the 3′- and the 5′-end, respectively (Fig. 2). The percentage of ‘rod-particles’ (shown in Figs. 3 to 5) contributing to the RNA-release intermediates was strongly enhanced by RNA crosslinking (Fig. 3); almost no (completely) empty particles were detected by CE (Fig. 6). Like FCS (Fig. 2), CE demonstrated the presence of externalized RNA in intermediate particles (Fig. 7). By using RT-PCR, only sequences close to the 5′-end of the RNA were detected in RNase-digested uncoating intermediates (Fig. 8), indicating that the 3′-end had become accessible.

In the present paper we demonstrate that RNA egress begins with the 3′-end. Our model is supported by two complementary findings. Firstly, we observed completely different kinetics for the exit of the 3′ and the 5′-ends of the RNA from the viral shell on incubation at 56°C. Deconvolution of the FCS autocorrelation data showed that the 3′-specific oligonucleotide first bound to molecular assemblies with diffusion properties expected for RNA that was still attached to the virus. At later time points, their diffusion rather corresponded to free RNA only, reflecting complete release from the virion. On the other hand, the oligonucleotide specific for the 5′-end never exhibited diffusion times corresponding to such virion-associated RNA. Secondly, in order to halt RNA transit at intermediate states, we used psoralen UV crosslinking. With capillary electrophoresis, we demonstrated that such crosslinked particles heated to 56°C indeed carried partially-externalized RNA that was accessible to a dsRNA-specific monoclonal antibody. We specifically digested this RNA, extracted the fraction that remained protected inside the virion, and, by RT-PCR, we exclusively found sequences close to the 5′-end. Fig. 9 shows our model of RNA release from HRV2 triggered by incubation at 56°C together with the results lending support to the model.

In the course of our experiments, we discovered that subviral particles containing ‘rod-like’ density were produced on incubation of native HRV2 at 56°C along with apparently full and empty virions. Similar uncoating intermediates with internal density taking on various conformations had also been observed in heated poliovirus [14]. We found that the particles originating from HRV2 were greatly enriched upon crosslinking double-stranded regions of the RNA prior to incubation at 56°C. This allowed for cryo-EM 3D-reconstruction, revealing that the rods were pointing approximately towards a two-fold axis and extended roughly towards a three-fold axis on the opposite side of the shell. The structural basis of this particular orientation needs further investigation. We do not know whether the orientation of the rod-like structure with respect to icosahedral symmetry is related to exit of the RNA – in the form of a single strand – through one of the channels at the two-fold axes. Nevertheless, it is conceivable, and supported by our findings, that the release process is initiated normally, i.e. the RNA starts to emerge through one of these pores, but gets stuck as soon as a double-stranded region cannot unwind. Such a scenario might lead to the build-up of rods remaining connected to the exit point that resemble RNA ‘prolate ellipsoids’ as observed in molecular dynamics simulations and small angle X-ray analysis of large RNA molecules [41].

Remarkably, upon closer analysis, the particles visually appearing to be full turned out to be a heterogeneous mixture of substantially distorted virions with a generally higher and broader distribution of RNA content (Fig. S2 and Table S1). This could indicate that RNA release from these particles was halted at an earlier stage as compared to the ‘rod-particles’. As previously observed in the presence of antiviral agents that inhibit breathing [42], impediment of ordered release may result in strain, causing partial denaturation/deformation of the capsid. In keeping with this hypothesis, the particles that had released more of the RNA (i.e. the psoralen crosslinks were closer to the 5′-end) were more homogenous as also indicated by a somewhat higher resolution attained in the 3DR despite smaller numbers of particle images (Fig. S2 and Table S1).

RNA detaching from the inner capsid wall, as is expected to occur on temperature elevation, may not be able to reform its original interactions upon cooling and thus be more prone to tangling. Alternatively, kinetic bottlenecks at regions of high secondary structures could be involved in the RNA condensation observed. ‘Rod-particles’ were only rarely observed on incubation of HRV2 at pH 5 in solution (data not shown); however, when HRV2 was bound to the surface of receptor-carrying liposomes and acidified some virions possessing internal density with a similar appearance were observed [19]. Thus, it is possible that membranes are involved in their formation at low pH, which will be a topic of future investigation. On transition from the native virion to the A-particle, a massive reorganisation of RNA-protein contacts is observed (unpublished data). These contacts might be essential for ordered release, possibly explaining why exposure to 56°C disturbs this tightly-coordinated process.

Ordered egress of RNA suggests that the viral genome becomes organized during packaging or assembly, which may occur co-transcriptionally [43], [44]. Therefore, it is likely that the process of encapsidation begins when the 5′-end emerges from the replication complex or at least before the complete RNA has been synthesized. It is also possible that the same applies to other viruses with ssRNA of positive polarity. This would imply that in these viruses, the 3′-end becomes encapsidated last, remaining near the capsid wall presumably in close proximity to one of the holes poised to open upon uncoating, thus resulting in a ‘last-in-first-out’ process of assembly and uncoating.

## Materials and Methods

### Materials

Unless otherwise indicated, all chemicals were acquired from Sigma Aldrich. 9-methoxy-7H-furo[3,2-g][1]benzopyran-7-on (i.e. 8-Methoxypsoralen, 8-MOP) was dissolved at 2 mg/ml in DMSO. Oligonucleotides (with and without fluorescent label) were obtained from VBC-Biotech, Vienna, Austria. Dylight 488 and FAM dye were from Thermo Scientific and dissolved in DMSO at 10 mg/ml. YOYO-1 iodide509 was from Life Technologies and dissolved in DMSO at 1 mM. Oligonucleotides for RT-PCR were as follows (denoted as under quotation marks)

“5′-end – reverse”

5′-dAAGGGTTAAGGTTAGCCACATTCAG-3′

“5′-end – forward”

5′-dGACCAATAGCCGGTAATCAG-3′

“ M-oligo – reverse”

5′-dAAGGTGTCAGTGTTATTTATTGGTACTAGGCTG-3′

“M-oligo – forward”

5′-dGCCCCATGTGTGCAGAGTTTTC-3′

“3′-end 3 - reverse”

5′-dCCACTCATGCAAAAGCAAATC-3′

“3′-end 3 - forward”

5′-dCCTTCCCTGAAGATAAATATTTGAATCC-3′

“3′-end 5 - reverse”

5′-dCTCTGGATCACATCCAACTGCTGATCCAG-3′

“3′-end 5 - forward”

5′-dGAGTTGACTTACCTATGGTCACC-3′

Their positions on the viral genome are indicated in the scheme in Fig. 8. All were chosen so as to have similar melting temperatures (50–58°C).

### Preparation of HRV2, psoralen UV crosslinking, virus and RNA fluorescence labeling

HRV2 was produced and purified as detailed elsewhere [11], [12] and incubated at ∼1.5 mg/ml with 8-MOP (50 µg/ml) for 4 h at 37°C in 50 mM Tris-HCl (pH 8.0). Samples were then pipetted onto parafilm on ice and irradiated with a UV lamp (Wilber Loumat, TFP-20L 6×15W) at 365 nm from a distance of about 5 cm for 10 min. Control samples were treated identically except that the preincubation with psoralen was omitted. Labeling of virus with DyLight 488 was performed following the protocol of the manufacturer. Viral RNA was transcribed *in vitro* by using the RiboMAX Large Scale RNA Production System – T7 (Promega) and purified by extraction with phenol chloroform followed by ethanol precipitation. For FCS measurements, *in vitro*-transcribed RNA was labeled with YOYO-1 iodide509; the dye was mixed with RNA (10 nM) at a molar ratio of 400∶1 (5 base pairs per dye molecule) in 50 mM Tris-HCl (pH 8.3), 75 mM KCl, 3 mM MgCl_2_ and incubated at room temperature for 15 min. With the exception of experiments involving RNase digestions, all samples were supplemented with 2U RNasin/µl (Promega).

### Fluorescence Correlation Spectroscopy (FCS)

FCS was carried out in a Zeiss Confocor 1 instrument using an Argon-ion laser with 488 nm wavelength. The fluorescence autocorrelation function was measured for the labeled oligonucleotides complementary to the HRV2 nucleotide numbers given in brackets: oligo-dT 25-mer (O3; between 7102 and ∼7200) and 5′-end (O5; 443–468) in the absence or presence of *in vitro*-transcribed viral RNA or heated HRV2, as indicated in the Figures. O5 was selected to be complementary to a region of low secondary structure by using the Vienna RNA package version 1.8 (http://rna.tbi.univie.ac.at/cgi-bin/RNAfold.cgi). Diffusion coefficients were related to the measured diffusion times by using Rhodamine6G as the standard (D_Rh6G_ = 2.8×10^−10^ m^2^ sec^−1^). For easier comparison, the autocorrelation functions were normalized to 1 at autocorrelation time zero (i.e. to one particle in the observation volume). Ten to 50 consecutive measurements on at least three independent samples were carried out with the respective fluorescent analytes at 10 nM (this gave the best signal to noise ratio) and a data acquisition time of 10 sec each, in 50 mM Tris-HCl (pH 8.3), 75 mM KCl, 3 mM MgCl_2_.

The most favorable molar ratios between oligonucleotide and RNA were determined by mixing labeled oligonucleotide at 10 nM with *in vitro*-transcribed HRV2 RNA at concentrations between 0 and 1000 nM (not shown). This revealed that at least a 25 fold excess (i.e. 250 nM) of heated virus/RNA over oligonucleotide was required for an optimal hybridization signal. The mixture was incubated for 10 min at 56°C and for at least 30 sec at 4°C prior to the measurments. To confirm that the observed change of the diffusion coefficient in the presence of viral RNA was due to hybridization, the sample was treated with a mixture of RNase A (1 mg/ml; Roche) and RNase H (0.5 U/µg RNA; New England BioLabs) for 15 min in 50 mM Tris-HCl (pH 8.3), 75 mM KCl, 3 mM MgCl_2_, 1 mM DTT at 37°C prior to the measurement (Fig. 1B, C). DyLight 488-labeled HRV2 was measured at 10 nM in 50 mM Tris buffer (pH 8.3). In all further experiments HRV2 at about 500 nM was mixed with 10 nM of the respective labeled oligonucleotide. The mixture was incubated at 56°C, samples (20 µl) were withdrawn at the times indicated in Fig. 2 and measured as detailed above. Diffusion times and percentages of free oligo, oligo bound to free RNA, and oligo bound to RNA partially released from the virus were calculated with the FCS ACCESS software (version 1.0.12) by using one-, two-, or three-component fit models as indicated. Hybridization equilibrium was reached almost instantaneously as deduced from the lack of change in the diffusion time within the measurement period (5 min).

For comparison with the measured values, diffusion coefficients of probe and viral RNA were calculated for hypothetic conformational states expected to result in lowest and highest diffusion coefficients (at 22°C, with the dynamic viscosity of water μ = 0.955×10^−3^ Pa·s). Assuming that they adopt a densely-packed sphere with mass density of 1.8 g/cm^3^ [45], the calculated diameters were 2.52 nm and 16.2 nm for probe and viral RNA respectively. In case of a rod-like shape, the probe (single-stranded DNA) was estimated to have a diameter of 1.1 nm and a length of 10.75 nm (25 bases, 0.43 nm length each), which will give the lowest diffusion coefficient [46]. For the viral RNA, an overall length of 3.85 µm (7102 bases, 0.43 nm each) and a persistence length of about 2 nm [47] were used and resulted in a calculated random coil diameter of 60 nm. For the viral particle, the diffusion coefficient was calculated on the basis of a perfect sphere with a radius of 15 nm. Table 1 lists those calculated values comparing them to the measured data.

### Capillary Electrophoresis

An automated HP3D Capillary Electrophoresis System (Hewlett Packard, Waldbronn, Germany) equipped with an uncoated fused-silica capillary (Composite Metal Service Ltd., 51.5-cm effective, 60.0-cm total length, 50 µm inner diameter) packed in a standard Hewlett Packard cassette and thermostated at 20°C was used throughout. Injection was at 50 millibar pressure for 9 sec. Between all runs the capillary was conditioned by aspirating 100 mM NaOH, water, and the background electrolyte (BGE, consisting of 100 mM Na-borate buffer (pH 8.3) and 10 mM Thesit) for 2 min each, applying 950 millibar pressure. Detector signals were recorded at 205 nm. For additional measurements at 260 nm, fast spectral scanning mode was employed. Positive polarity mode (negative pole is placed at the capillary outlet) with 25 kV was used for all experiments. HRV2, either psoralen crosslinked or not, was incubated at 56°C for different times as indicated in the Figures. To detect viral RNA outside the virion but still connected to it, crosslinked virus either heat-treated or not (control) was incubated with 1 µl (1 µg)/sample anti-dsRNA mAB J2 (English & Scientific Consulting Bt. Szirák, Hungary) for 20 min at room temperature. To remove accessible RNA, crosslinked and heat-treated (10 min at 56°C) virus was incubated with micrococcal nuclease (MNase, 100 units/µg RNA; New England BioLabs) at 37°C for 20 min. MNase was inactivated by addition of 10 mM EGTA. Subviral particles were quantified as total protein by integration of the peak area at 205 nm and subtraction of the respective calibrated peak area at 260 nm (viral protein = peak area (at 205 nm)−1.4×peak area (at 260 nm)). The RNA content of the particles was estimated by comparison with native virus (set to 100%); peak areas were recorded at 205 nm and 260 nm for native virus from 12 different preparations giving a ratio of 5.5±0.1 and after subtraction of the contribution of VP4 of 5.3±0.1 (i.e. full 135S particles). For crosslinked, heated, and MNase-digested particles, this ratio was 12.6±1.0 (4 different electropherograms). Based on a ratio of the extinction at 205 nm/260 nm for pure RNA of 1.4, the mean RNA content of these latter particles was estimated to amount to 34.7%.

Assuming irreversible release of the RNA (irreversible mass action), the evolution of the concentrations of the three components in time was modelled by using GEPASI v. 3.30 [48] based on a reaction model N→V−R→R+V (shown as lines in Fig. 6).

### Determination of RNA sequences protected from nuclease

Crosslinked HRV2 either incubated at 56°C for 10 min or not (control) was MNase treated as above and run on a 0.7% agarose gel in 50 mM Tris-HCl (pH 8.3), 10 mM EDTA. RNA was visualized by ethidium bromide-staining and extracted from the bands with the Zymoclean Gel RNA Recovery Kit (Zymo Research). Reverse transcription (RT) was carried out with SuperScript III reverse transcriptase (Invitrogen). PCR amplification was performed with *Pfu* DNA polymerase (Promega) by using the primer sets given above for 30 cycles. For protein analysis, aliquots of the excised bands were boiled in reducing sample buffer and run on a SDS polyacrylamide gradient gel (12–20%). Proteins were detected by silver staining [49].

### Electron microscopy and 3D image reconstruction

Psoralen UV crosslinked HRV2 was incubated at 56°C for 10 min. Negative stain EM was carried out at 56,000× magnification (80 kV) in a FEI Morgagni 268D equipped with an 11 megapixel CCD camera (Morada from Olympus-SIS) after staining with 2% phosphotungstic acid. For cryo-EM, samples (4 µl) were deposited on Quantifoil 400 nm copper grids with a 1.2/1.3 holey carbon film (glow discharged in a Bal-Tec SCD Sputter Coater (20 mA, 1 min; Scotia)), blotted with an automatic Leica EM Grid Plunger, and shock frozen in liquid ethane cooled in liquid nitrogen. Image acquisition was done as follows: Subviral particles were viewed in a FEI Tecnai F30 Polara cryo-electron microscope (300 kV) with C2 condenser aperture and objective aperture of 70 µm and 100 µm, respectively, at a magnification of 71,949 x. Images were acquired at underfocus between 2.0 µm and 4.2 µm with a Gatan Ultrascan 4000 4k CCD camera, using Leginon automatic image acquisition software [50]. The micrographs (383 finally used) were corrected for the CTF with ctffind3 [51] and downscaled to 128×128 pixels at 3.76 Å/pixel by using xmipp 2.4 [52], [53]. 3D-maximum likelihood classification into three classes was done with relion-1.1 [54] on 16,151 particle images (downscaled to 64×64 pixel) by using the cryo-EM 3DR of full HRV2 A-particles (unpublished data) as starting map. On using an angular sampling interval of 15°, 4581 images were classified as full particles, 5285 images as ‘rod-containing’ particles, and 6285 images as empty particles. Final maps were then refined by using particle images with 128×128 pixels as selected in the previous classification, with relion-1.0 with and without imposing symmetry as specified in the figures [54]. The resolution of the asymmetric 3DR (between 22 and 24 Å, as determined from the spectral signal to noise ratio - SSNR∧MAP>1; see ref. [54]) was sufficient to identify the orientation of the ‘rod’ with respect to icosahedral symmetry. The RNA content was estimated for each (centered) image after normalization to mean = 0 and standard deviation = 1 by using xmipp-2.4. For each class, the density corresponding to the RNA (within a radius of 113 Å i.e. 0–15 pixels on the 64×64 pixel images) was related to part of the density of the protein shell (between radius 113 and 143 Å (i.e. 15–19 pixels; see Fig. S1) and displayed as a histogram plot.

## Supporting Information

Figure S1 Spherically averaged radial density plots of 3DR of the three HRV2 subviral particle classes selected by maximum likelihood 3D classification. Radii delimiting the RNA core and the protein shell respectively, as used for the calculation of the relative core density (Fig. 4B), are indicated with broken lines.
(TIF)

Figure S2 Comparison of various native and subviral enterovirus particles with HRV2 subviral particles as obtained on heating to 56°C for 10 min. A) Volumes were computed with bgex [1], [2] from the X-ray coordinates as indicated (HRV2 native, 1fpn; B-particle, 3tn9; EV71 native, 3vbs; B-particle, 3vbr; PV native, 2plv). For poliovirus a cryo-EM model of the 135S subviral A-particle (EMD-1133) is shown instead. All volumes were filtered to 14 Å (the highest resolution obtained in our reconstructions) and displayed as radially color-coded surfaces. B) 3DR of HRV2 subviral particles (from 16,151 images downscaled to 64×64 pixels) were subjected to ML3D classification with relion-1.1 [3], [4], [5] into 10 classes without imposing symmetry by using the cryo-EM map of the 135S subviral A-particle (unpublished data) as a starting map. The number of particle images combined in each class is summarized in Table S1. Four classes were populated each with less than 5% of all particle images and not considered further; examination of the corresponding images revealed a heterogeneous population of substantially deformed virions. The 6 remaining classes obtained from ML3D were then refined with relion-1.0 using images with 128×128 pixels either imposing icosahedral symmetry, upper row; or without imposing symmetry, middle row, and rendered as radially color-coded surfaces. Central sections are displayed in the lower row. All volumes are viewed down a 2-fold axis at sigma = 1 above the mean density except from the ‘rod-containing particles’ that are displayed at sigma = 1.7 (class2); 2.0 (class3); 1.5 (class4), for better appreciation of the preferred contact site of the ‘rod’ with the inner wall of the protein shell (close to a 2-fold axis). The respective resolutions are summarized in Table S1. Note the obvious deviations from icosahedral symmetry in the class1 particle following asymmetric reconstruction.
(TIF)

References S1 Supporting information references.
(DOCX)

Table S1 Summary of the 6 ML3D-classes of subviral HRV2 particles that represented more than 5% of the total number of particle images. The resolution of the respective reconstructions refers to the spectral signal to noise ratio (SSNR∧MAP>1) see [5]. The ‘degree of symmetry’ was estimated from the correlation, determined with Chimera [6], between the reconstructions made with (I2) and without (C1) imposing icosahedral symmetry after masking the RNA and Fourier filtering to 30 Å resolution.
(DOCX)
