# Supplementary figures and images for: Viral Uncoating Is Directional: Exit of the Genomic RNA in a Common Cold Virus Starts with the Poly-(A) Tail at the 3′-End
Source: PLoS Pathog. 2013 Apr 4;9(4):e1003270. doi: 10.1371/journal.ppat.1003270 (PMC3617019; doi:10.1371/journal.ppat.1003270)

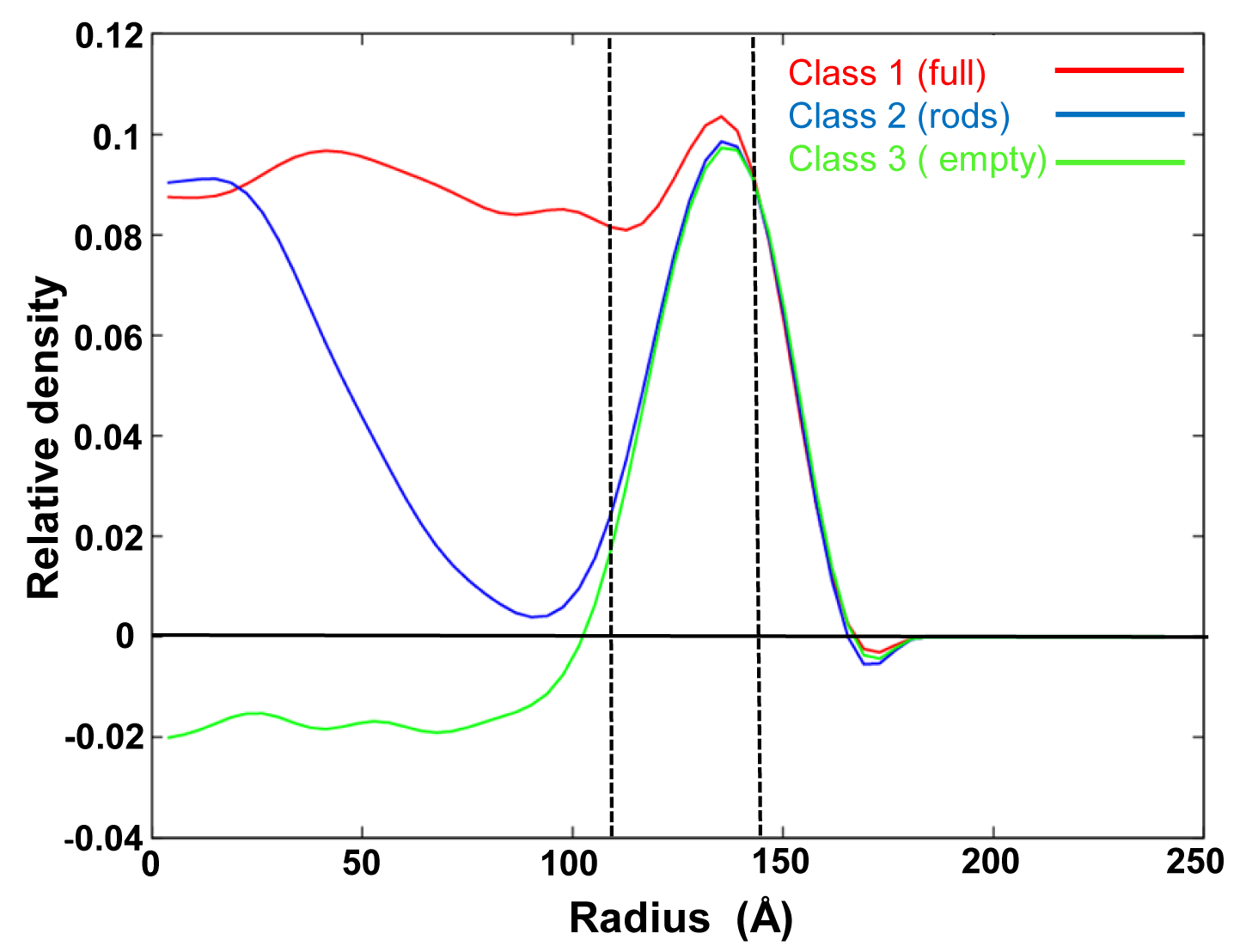

Supplement: Figure S1 — Spherically averaged radial density plots of 3DR of the three HRV2 subviral particle classes selected by maximum likelihood 3D classification. Radii delimiting the RNA core and the protein shell respectively, as used for the calculation of the relative core density (Fig. 4B), are indicated with broken lines. (TIF) [file ppat.1003270.s001.tif]

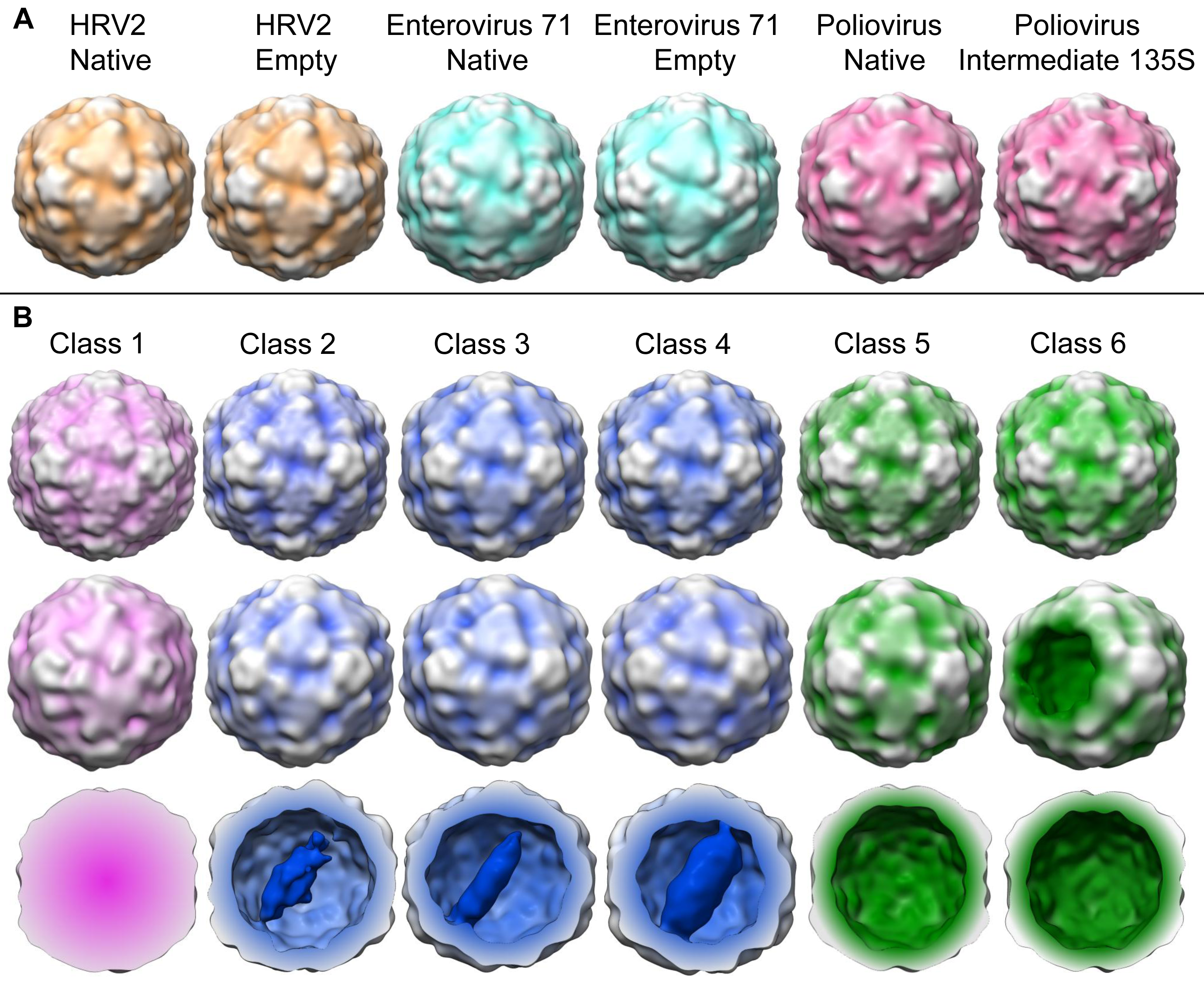

Supplement: Figure S2 — Comparison of various native and subviral enterovirus particles with HRV2 subviral particles as obtained on heating to 56°C for 10 min. A) Volumes were computed with bgex [1], [2] from the X-ray coordinates as indicated (HRV2 native, 1fpn; B-particle, 3tn9; EV71 native, 3vbs; B-particle, 3vbr; PV native, 2plv). For poliovirus a cryo-EM model of the 135S subviral A-particle (EMD-1133) is shown instead. All volumes were filtered to 14 Å (the highest resolution obtained in our reconstructions) and displayed as radially color-coded surfaces. B) 3DR of HRV2 subviral particles (from 16,151 images downscaled to 64×64 pixels) were subjected to ML3D classification with relion-1.1 [3], [4], [5] into 10 classes without imposing symmetry by using the cryo-EM map of the 135S subviral A-particle (unpublished data) as a starting map. The number of particle images combined in each class is summarized in Table S1. Four classes were populated each with less than 5% of all particle images and not considered further; examination of the corresponding images revealed a heterogeneous population of substantially deformed virions. The 6 remaining classes obtained from ML3D were then refined with relion-1.0 using images with 128×128 pixels either imposing icosahedral symmetry, upper row; or without imposing symmetry, middle row, and rendered as radially color-coded surfaces. Central sections are displayed in the lower row. All volumes are viewed down a 2-fold axis at sigma = 1 above the mean density except from the ‘rod-containing particles’ that are displayed at sigma = 1.7 (class2); 2.0 (class3); 1.5 (class4), for better appreciation of the preferred contact site of the ‘rod’ with the inner wall of the protein shell (close to a 2-fold axis). The respective resolutions are summarized in Table S1. Note the obvious deviations from icosahedral symmetry in the class1 particle following asymmetric reconstruction. (TIF) [file ppat.1003270.s002.tif]
