# Supplementary material for: Viral Uncoating Is Directional: Exit of the Genomic RNA in a Common Cold Virus Starts with the Poly-(A) Tail at the 3′-End
Source: PLoS Pathog. 2013 Apr 4;9(4):e1003270. doi: 10.1371/journal.ppat.1003270 (PMC3617019; doi:10.1371/journal.ppat.1003270)
Supplement: References S1 — Supporting information references. (DOCX) [file ppat.1003270.s003.docx]

1. Heymann JB, Belnap DM (2007) Bsoft: image processing and molecular modeling for electron microscopy. J Struct Biol 157: 3-18.

2. Heymann JB (2001) Bsoft: image and molecular processing in electron microscopy. J Struct Biol 133: 156-169.

3. Sigworth FJ, Doerschuk PC, Carazo JM, Scheres SH (2010) An introduction to maximum-likelihood methods in cryo-EM. Methods Enzymol 482: 263-294.

4. Scheres SH (2010) Classification of structural heterogeneity by maximum-likelihood methods. Methods Enzymol 482: 295-320.

5. Scheres SH (2012) A Bayesian view on cryo-EM structure determination. J Mol Biol 415: 406-418.

6. Pettersen EF, Goddard TD, Huang CC, Couch GS, Greenblatt DM, et al. (2004) UCSF Chimera - a visualization system for exploratory research and analysis. J Comput Chem 25: 1605-1612.
