# Supplementary material for: Viral Uncoating Is Directional: Exit of the Genomic RNA in a Common Cold Virus Starts with the Poly-(A) Tail at the 3′-End
Source: PLoS Pathog. 2013 Apr 4;9(4):e1003270. doi: 10.1371/journal.ppat.1003270 (PMC3617019; doi:10.1371/journal.ppat.1003270)
Supplement: Supplementary file 3 [file ppat.1003270.s004.docx]

	Class 1 (Full)	Class 2 (Rods)	Class 3 (Rods)	Class 4 (Rods)	Class 5 (Empty)	Class 6 (Empty)
Number of Particles	4227	2900	1989	1316	3037	1724
Resolution (Å) (I2)	15	16	17	16	14	18
Resolution (Å) (C1)	25	27	28	30	25	30
Correlation C1/I2	0.90	0.96	0.93	0.96	0.97	0.91
